# Supplementary material for: Expression Characteristics in Roots, Phloem, Leaves, Flowers and Fruits of Apple circRNA
Source: Genes (Basel). 2022 Apr 18;13(4):712. doi: 10.3390/genes13040712 (PMC9030095; doi:10.3390/genes13040712)
Supplement: Supplementary file 1 [file genes-13-00712-s001.zip › Figure S.pdf]

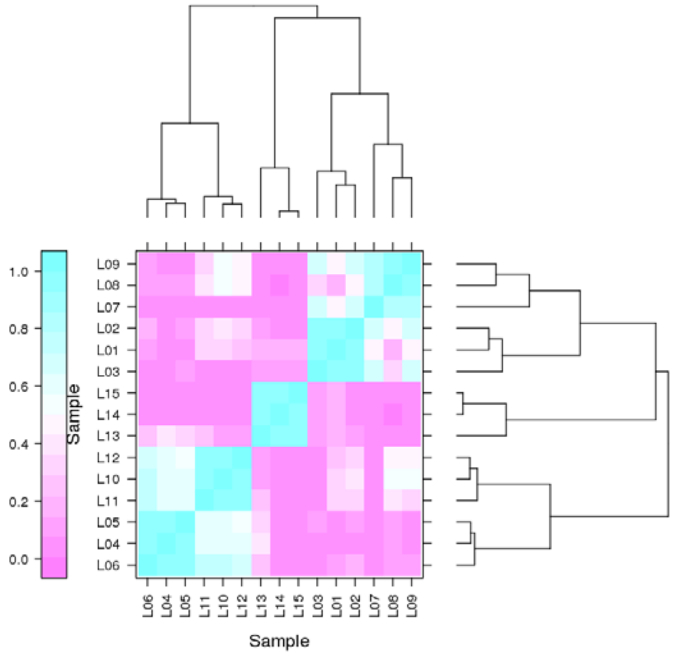

Figure S1. Heatmap of pearson's correlation coefficient between replicates of samples

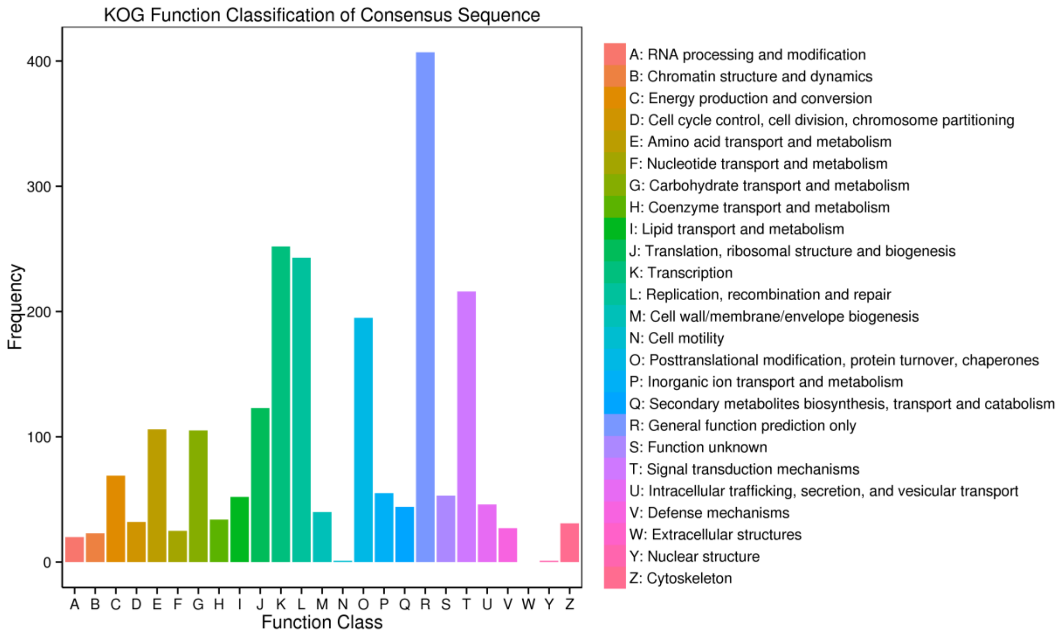

Figure S2. Cluster of genes (KOG) classifications of host-genes (protein-based genes) of circRNAs.
